# Supplementary material for: Mechanically strong MXene/Kevlar nanofiber composite membranes as high-performance nanofluidic osmotic power generators
Source: Nat Commun. 2019 Jul 2;10:2920. doi: 10.1038/s41467-019-10885-8 (PMC6606750; doi:10.1038/s41467-019-10885-8)
Supplement: Supplementary file 1 — Supplementary Information [file 41467_2019_10885_MOESM1_ESM.pdf]

Supplementary Information

for

Mechanically Strong MXene/Kevlar Nanofiber Composite Membranes as High-Performance Nanofluidic Osmotic Power Generators

Zhen Zhang, Sheng Yang, Panpan Zhang, Jian Zhang, Guangbo Chen and Xinliang Feng*

Center for Advancing Electronics Dresden (Cfaed) and Department of Chemistry and Food Chemistry, Technische Universität Dresden, 01062 Dresden, Germany

*Corresponding author. E-mail: xinliang.feng@tu-dresden.de

Supplementary Note 1 | Electrode Calibration

The osmotic energy conversion is investigated by measuring the *I-V* curves in presence of a transmembrane concentration gradient. The sweeping voltage was from -0.4 V to 0.4 V with a step of 0.04 V. The experimental setup can be represented in termed of the equivalent circuit shown in Supplementary Fig. 8. E_{mea}, E_{redox}, E_{diff}, and R_{o} represent the measured potential, the redox potential generated by the unequal potential drop at the electrode–solution interface, the diffusion potential contributed by the ion selective membrane, and the inner resistance of the membrane, respectively. The measured E_{mea} actually consists of two parts (i.e. E_{redox} and E_{diff}).[1] In this work, the value of E_{redox} was measured using an experimental method. The separator membrane was replaced by a nonselective silicon membrane containing a single micro-window. In this case, the measured potential was contributed solely by the asymmetric redox reactions on the electrodes (E_{redox}). The electrode potential remained stable during the calibration process as the diffusion of ions did not affect the bulk concentration obviously in the first several minutes. Such an experimental method can largely preclude the influence brought by many unexpected factors such as the contamination and electrode imperfection.[2-4] Supplementary Table 2 shows the values of E_{mea}, E_{redox}, and E_{diff}.

Supplementary Note 2 | Energy Conversion Efficiency

For a given concentration gradient, the cation transference number (i.e. t_+) can be calculated as:

$$t_+ = \frac{1}{2}\left(\frac{E_{diff}}{\frac{RT}{zF}\,\mathrm{In}\left(\frac{r_{c_H} c_H}{r_{c_L} c_L}\right)} + 1\right)$$

Here, E_{diff}, R, T, F, z, γ, and c refer to the diffusion potential, universal gas constant, absolute temperature, Faraday constant, charge number, activity coefficient of ions, ion concentration, respectively. The energy conversion efficiency can be defined as the ratio of the output energy (electrical energy) to the input energy (Gibbs free energy of mixing). Accordingly, the energy conversion efficiency corresponding to the

maximum power generation can be calculated as[4, 5]:

$$\eta_{\max} = \frac{1}{2}(2t_{+} - 1)^2$$

Supplementary Note 3 | Numerical Simulation

The synergetic effect of space charge and surface charge is theoretically investigated using a commercial finite-element software package COMSOL Multiphysics. The "electrostatics (Poisson equation)" and "Nernst-Planck without Electroneutrality" modules are selected.

The flux equations for each ionic species contributed by the concentration gradient induced diffusion current and potential gradient induced drift current can be physically described by the Nernst-Planck equation[6, 7]:

$$\mathbf{J}_{\mathrm{i}} = D_{\mathrm{i}}(\nabla c_{\mathrm{i}} + \frac{z_{\mathrm{i}} F c_{\mathrm{i}}}{RT}\nabla\varphi) + c_{\mathrm{i}}\mathbf{u} \quad , \quad (\mathrm{i} = +1 \text{ or } -1) \qquad (1)$$

where $\mathbf{J}_i$, D_i, c_i, φ, $\mathbf{u}$, R, F, and T refer to the ionic flux, diffusion coefficient, ion concentration, electrical potential, fluid velocity, universal gas constant, Faraday constant, and absolute temperature, respectively. The relationship between the electric potential and ion concentrations satisfies the Poisson equation:

$$-\nabla^2\varphi = \frac{f\rho_{\mathrm{s}} + \rho_0}{\varepsilon} \quad , \quad (f = 1 \text{ or } 0) \qquad (2)$$

where ε refer to dielectric constant of the electrolyte solutions, ρ_o is the space charge density of the mobile ions ($\rho_o = F \sum_i z_i c_i$), ρ_s is the space charge brought by ANF. When the system reaches a stationary regime, the ionic flux should satisfy the steady-state equation:

$$\nabla \cdot \mathbf{J}_{\mathrm{i}} = 0 \qquad (3)$$

The couple equations are solved neglecting hydrodynamic effects (**u**=0) and assuming appropriate boundary conditions. A sketch of the computation domain is shown in Supplementary Fig. 13. In order to gain affordable computation scale, the fluidic pathway through the composite membrane is simplified to be a 1000 nm long single 2D channel. As the interlayer spacing is largely expanded by ANF, the size of the channel is set to be 10 nm, consistent with the experimentally measured diameter

of the nanofiber. Two electrolyte reservoirs (400 $\times$ 200 nm) were added to minimize the influence of the resistance of mass transfer at the entrance and exit. Two surfaces (W1 and W2) correspond to the electrodes used to apply a voltage across the channel. The ion flux has the zero normal components at boundaries:

$$\mathbf{n}\cdot\mathbf{J}_\mathrm{i}=0 \qquad (4)$$

In this work, the surface charge and space charge is set to be within reasonable ranges: in the same order of magnitude with the charge density of metal oxide/carbide and polymer-brushes filled nanochannel. The concentration of the electrolyte in the two reservoirs is set to 50-fold (i.e. 0.5 M/0.01 M).

The ionic current through the nanochannel can be calculated:

$$I=\int_s F(z_\mathrm{p}\mathbf{J}_\mathrm{p}+z_\mathrm{n}\mathbf{J}_\mathrm{n})\cdot\mathbf{n}\mathrm{d}S \qquad (5)$$

By varying the external potential from -0.4 V to 0.4 V, a theoretical current-voltage curve can be obtained. Accordingly, the open-circuit voltage and short-circuit current can be derived from the intercept on X/Y axis. The corresponding power output and energy conversion efficiency can be calculated. The detailed simulation parameter is shown in Supplementary Table 3.

Supplementary Figures and Tables

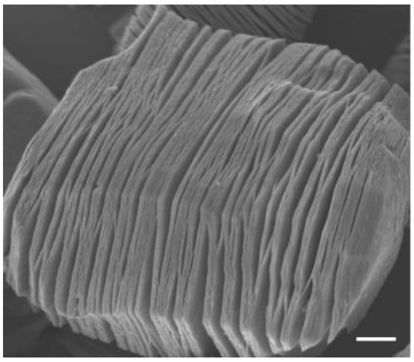

Supplementary Figure 1. SEM images synthesized accordion-like MXene powder (scale-bar: 400 nm).

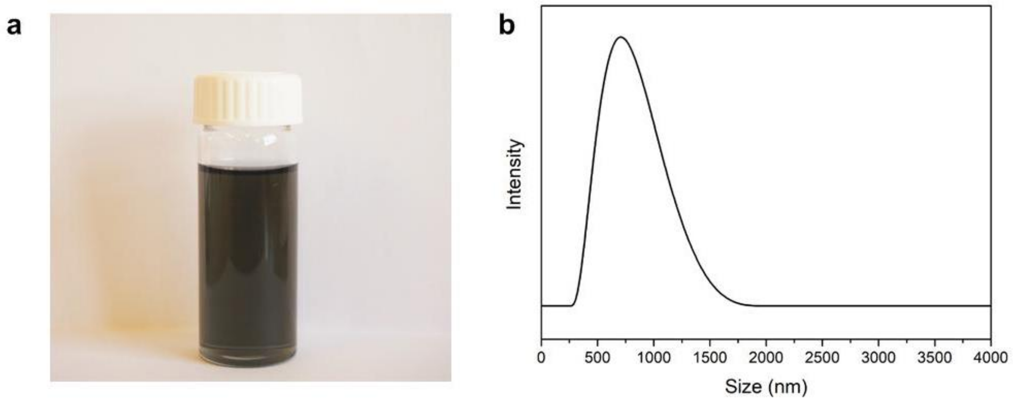

Supplementary Figure 2. (a) Photograph of $Ti_3C_2T_x$ dispersion in DMSO. (b) Size distribution of the as-prepared $Ti_3C_2T_x$.

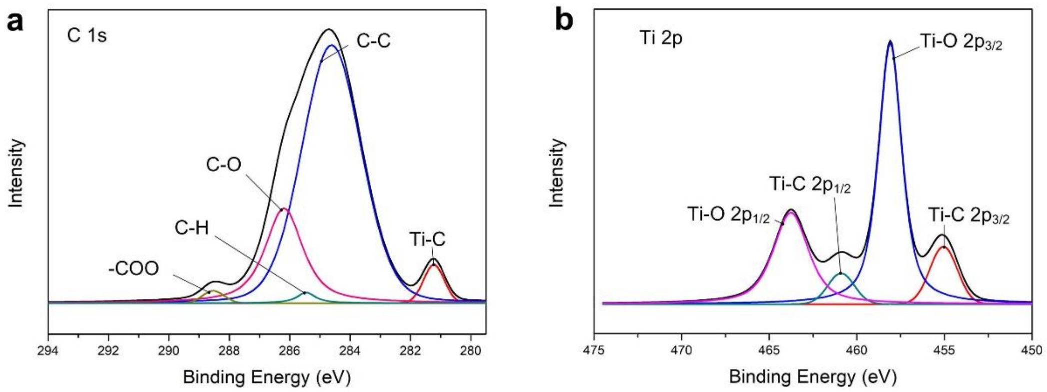

Supplementary Figure 3. High resolution C 1s (a) and Ti 2p (b) XPS spectra of the as-prepared MXene, indicating presence of C-O, Ti-O bonds from the surface groups.

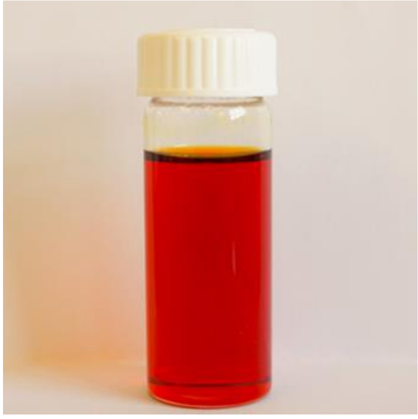

Supplementary Figure 4. Photograph of ANF dispersion in DMSO.

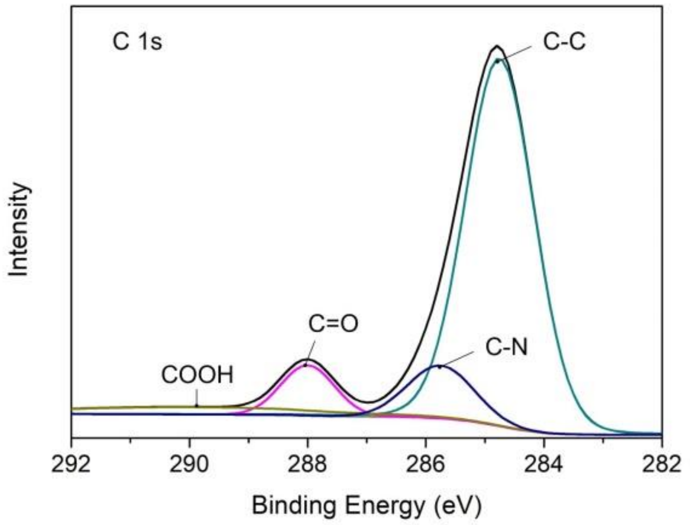

Supplementary Figure 5. C 1s XPS spectra of the ANF, indicating the existence of surface functional carboxyl groups.

Bond	C-C	C−N	C=O	COOH
Bind Energy (eV)	284.7	285.7	287.9	289.9
Area (%)	76.08	10.22	7.82	5.88

Supplementary Table 1. Bonding-state peak locations and concentrations of the decomposed C 1s energy state of the ANF.

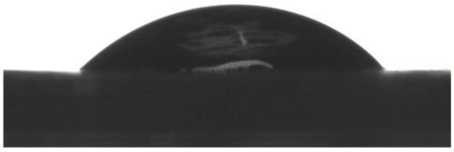

Supplementary Figure 6. Wettability of the composite membrane. The as-prepared composite membrane is hydrophilic with a contact angle about 45°.

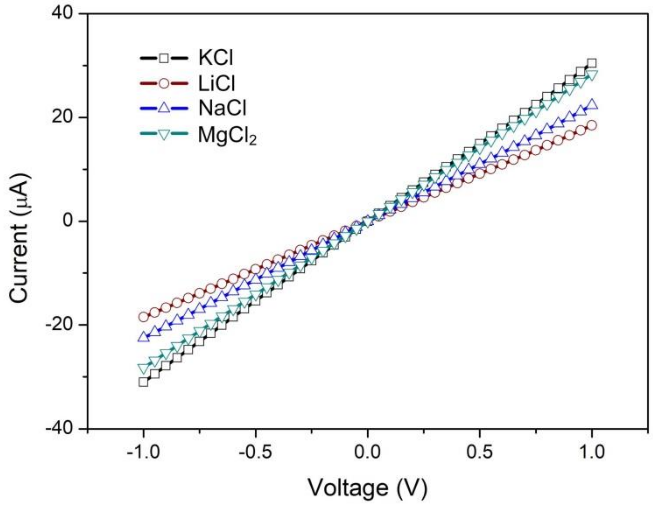

Supplementary Figure 7. *I-V* curves of composite membrane recorded in neutral 0.1 M solution of different electrolyte. Besides Li^+, Na^+, and K^+ ions, the membrane also behaves large conductance towards Mg^{2+} ion.

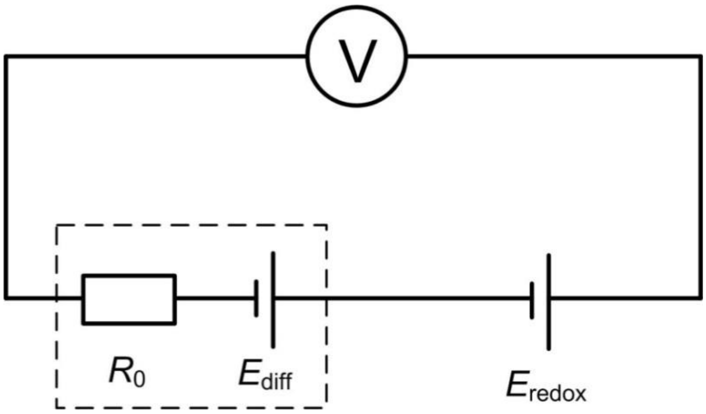

Supplementary Figure 8. The equivalent circuit of the membrane based power source.

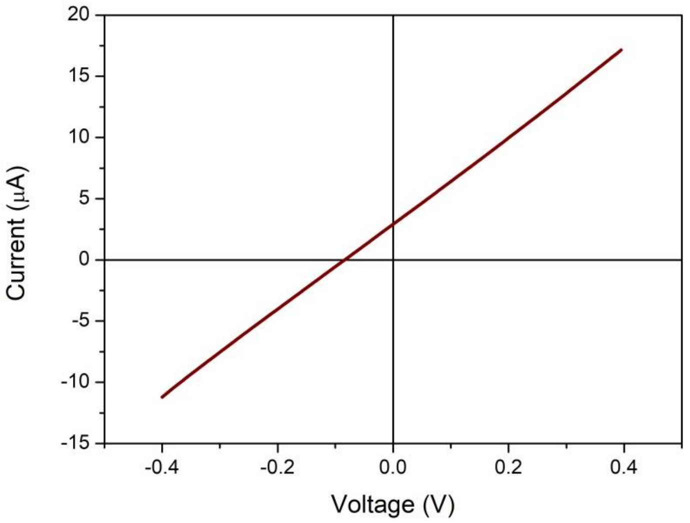

Supplementary Figure 9. *I-V* of the composite membrane recorded in 0.5 M/0.01 M NaCl salinity gradient after subtracting the contribution of redox potential.

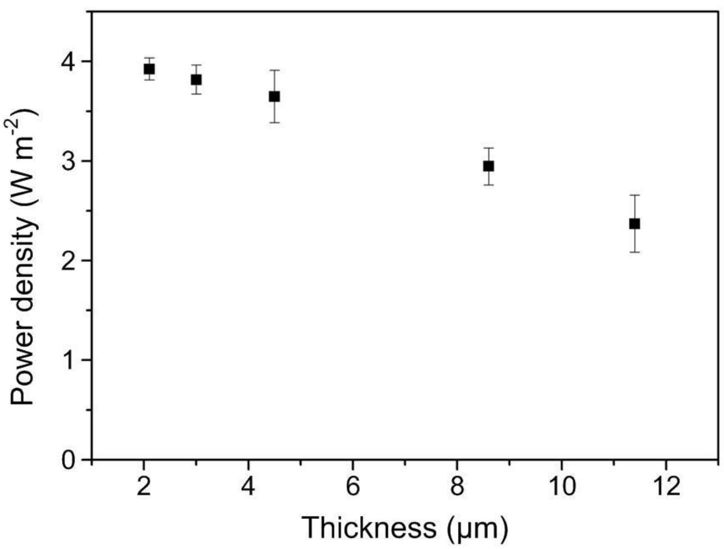

Supplementary Figure 10. Influence of the thickness of the composite membrane (ANF content: 11 %) on the power density. Error bars represent s.d.

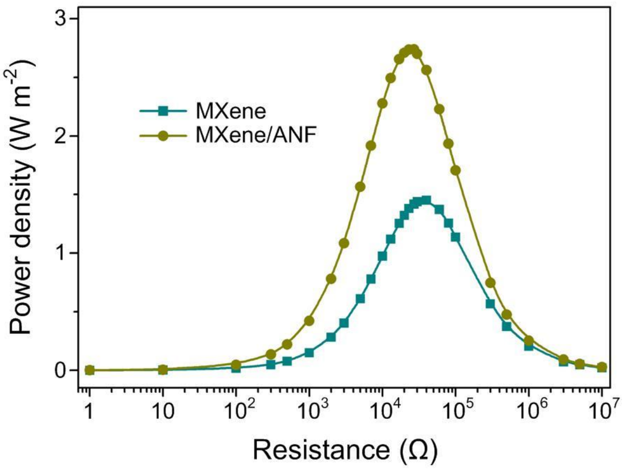

Supplementary Figure 11. Comparison of the power density of pristine MXene and MXene/ANF composite membranes. The power density of composite system (2.74 W m^{-2}) is much larger than that of the pristine system (1.44 W m^{-2}), indicating the enhancing effect of the nanofiber on the energy conversion performance. Here, the pristine MXene and MXene/ANF (11%) are both supported by a non-functional porous hydrophilic nylon-66 membranes (pore size~0.2 μm). Notably, the power density is lower than that of unsupported samples as the supporting membrane will introduce extra fluid resistance.

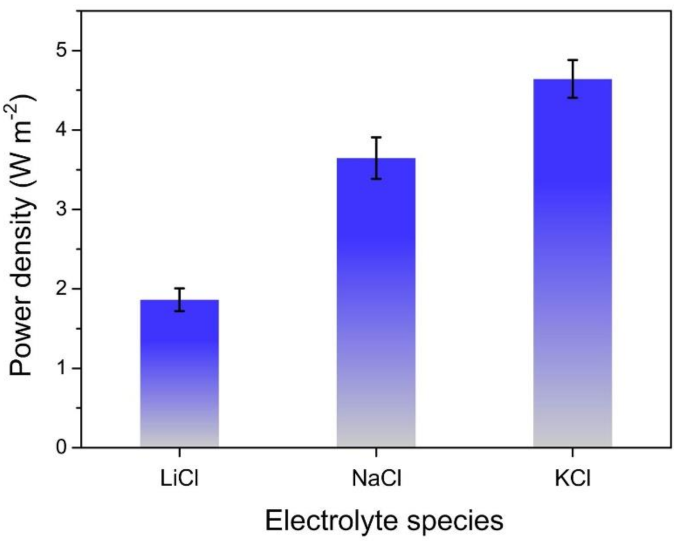

Supplementary Figure 12. The dependence of the electrolyte species on the output power density. Error bars represent s.d.

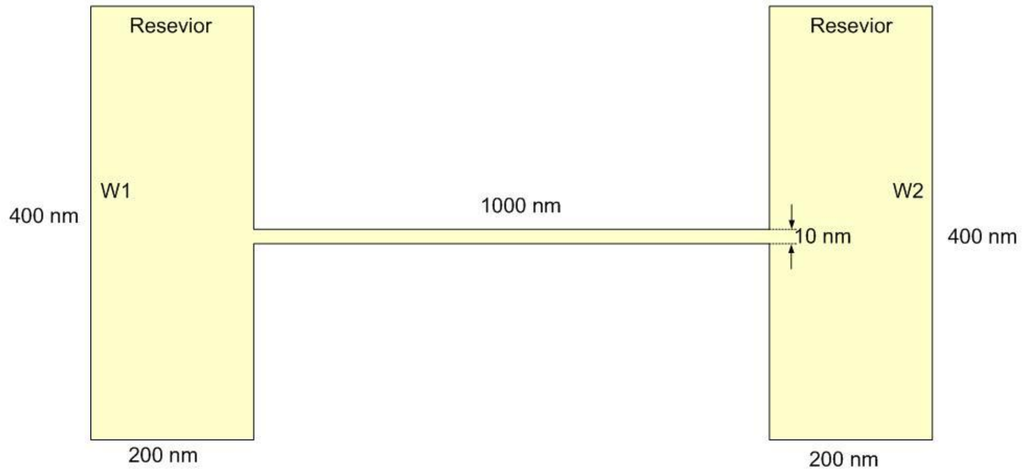

Supplementary Figure 13. Model of theoretical simulation.

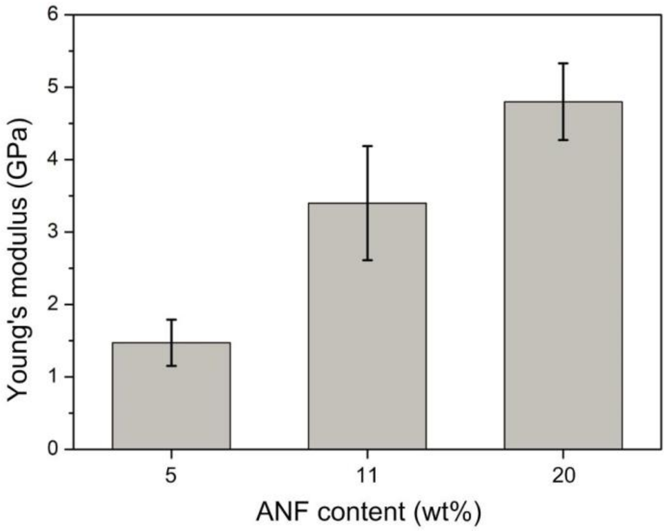

Supplementary Figure 14. Young’s modulus of the MXene/ANF composite membrane with different ANF content. Error bars represent s.d.

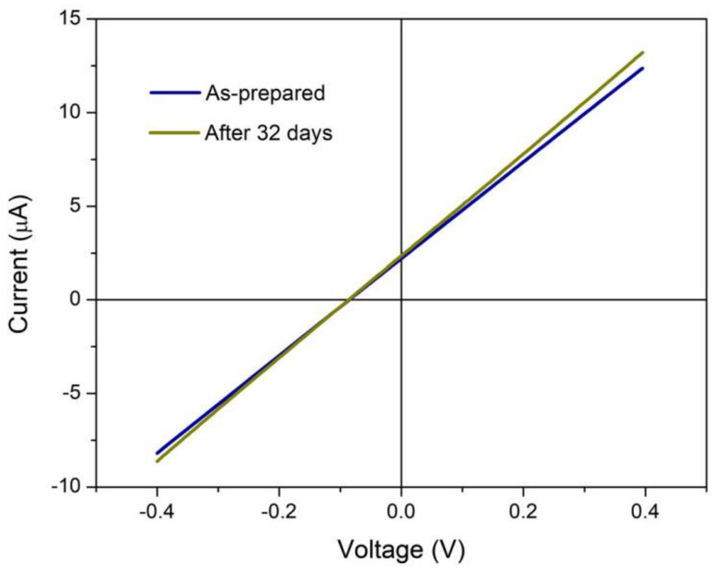

Supplementary Figure 15. *I-V* of the composite membrane recorded in river water/sea water system after subtracting the contribution of redox potential.

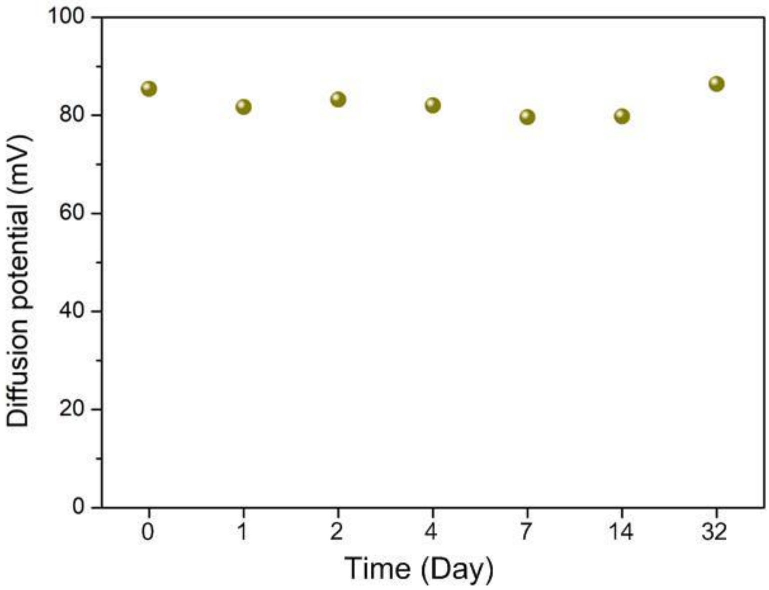

Supplementary Figure 16. The diffusion potential as a function of time.

	10-fold	50-fold	100-fold	250-fold	1000-fold
E_{mea} (mV)	34.06	91	122.6	158.36	203.8
E_{redox} (mV)	22.2	48.8	66	76.4	103
E_{diff} (mV)	11.86	42.2	56.6	81.96	100.8

Supplementary Table 2. List of E_{mea}, E_{redox}, and E_{diff} (the low concentration side is set to be 0.1 mM KCl).

Surface charge ($C\ m^{-2}$)	Space charge ($C\ cm^{-3}$)	I_{SC} (A)	V_{OC} (mV)	Surface charge ($C\ m^{-2}$)	Space charge ($C\ cm^{-3}$)	I_{SC} (A)	V_{OC} (mV)
-0.001	0	0.0000003	0.1	-0.01	-12	0.00142	56.8
-0.005	0	0.000115	5.9	-0.01	-16	0.0017	65.4
-0.01	0	0.00026	12.4	-0.02	-4	0.001	42.9
-0.02	0	0.00053	24.5	-0.02	-8	0.00136	55.7
-0.03	0	0.00078	34.3	-0.02	-12	0.00166	65.6
-0.04	0	0.00101	43	-0.02	-16	0.00194	73.6
-0.05	0	0.00125	52.2	-0.03	-4	0.00123	51.4
0	-0.2	0.0000043	0.00014	-0.03	-8	0.00159	63.8
0	-1	0.00011	5.3	-0.03	-12	0.0019	73.6
0	-4	0.00049	22.7	-0.03	-16	0.00218	81.7
0	-8	0.00089	38.7	-0.04	-0.2	0.00104	43.7
0	-12	0.00122	49.8	-0.04	-1	0.00113	47.5
0	-16	0.00151	59.6	-0.04	-4	0.00144	58.8
0	-20	0.00178	67.9	-0.04	-8	0.00179	70.6
-0.001	-4	0.00051	24	-0.04	-12	0.00209	79.9
-0.005	-4	0.00061	28	-0.04	-16	0.00238	88
-0.01	-4	0.00074	32.8	-0.04	-20	0.00264	95
-0.01	-8	0.00111	46.2	-0.05	-4	0.00164	66.4

Supplementary Table 3. Detailed simulation parameter in Fig. 5.

Supplementary References

1. Feng, J. *et al.* Single-layer MoS_2 nanopores as nanopower generators. *Nature* **536**, 197–200 (2016).
2. Choi, E., Kwon, K., Kim, D. & Park, J. Tunable reverse electrodialysis microplatform with geometrically controlled self-assembled nanoparticle network. *Lab Chip* **15**, 168–178 (2015).
3. Gao, J. *et al.* High-performance ionic diode membrane for salinity gradient power generation. *J. Am. Chem. Soc.* **136**, 12265–12272 (2014).
4. Ouyang, W., Wang, W., Zhang, H., Wu, W. & Li, Z. Nanofluidic crystal: a facile, high-efficiency and high-power-density scaling up scheme for energy harvesting based on nanofluidic reverse electrodialysis. *Nanotechnology* **24**, 345401 (2013).
5. Kim, D. K., Duan, C. H., Chen, Y. F. & Majumdar, A. Power generation from concentration gradient by reverse electrodialysis in ion-selective nanochannels. *Microfluid. Nanofluid.* **9**, 1215–1224 (2010).
6. Cheng, C., Jiang, G., Simon, G. P., Liu, J. Z. & Li, D. Low-voltage electrostatic modulation of ion diffusion through layered graphene-based nanoporous membranes. *Nat. Nanotechnol.* **13**, 685–690 (2018).
7. White, H. S. & Bund, A. Ion current rectification at nanopores in glass membranes. *Langumir* **24**, 2212–2218 (2008).
